# ProteoMutaMetrics: machine learning approaches for solute carrier family 6 mutation pathogenicity prediction†

**DOI:** 10.1039/d4ra00748d

**Published:** 2024-04-22

**Authors:** Jiahui Huang, Tanja Osthushenrich, Aidan MacNamara, Anders Mälarstig, Silvia Brocchetti, Samuel Bradberry, Lia Scarabottolo, Evandro Ferrada, Sergey Sosnin, Daniela Digles, Giulio Superti-Furga, Gerhard F. Ecker

**Affiliations:** a University of Vienna, Department of Pharmaceutical Sciences Vienna Austria gerhard.f.ecker@univie.ac.at; b Bayer AG, Division Pharmaceuticals, Biomedical Data Science II Wuppertal Germany; c Emerging Science & Innovation, Pfizer Worldwide Research, Development and Medical Cambridge MA USA; d Axxam SpA Bresso Milan Italy; e CeMM, Research Center for Molecular Medicine of the Austrian Academy of Sciences Vienna Austria

## Abstract

The solute carrier transporter family 6 (SLC6) is of key interest for their critical role in the transport of small amino acids or amino acid-like molecules. Their dysfunction is strongly associated with human diseases such as including schizophrenia, depression, and Parkinson's disease. Linking single point mutations to disease may support insights into the structure–function relationship of these transporters. This work aimed to develop a computational model for predicting the potential pathogenic effect of single point mutations in the SLC6 family. Missense mutation data was retrieved from UniProt, LitVar, and ClinVar, covering multiple protein-coding transcripts. As encoding approach, amino acid descriptors were used to calculate the average sequence properties for both original and mutated sequences. In addition to the full-sequence calculation, the sequences were cut into twelve domains. The domains are defined according to the transmembrane domains of the SLC6 transporters to analyse the regions' contributions to the pathogenicity prediction. Subsequently, several classification models, namely Support Vector Machine (SVM), Logistic Regression (LR), Random Forest (RF), and Extreme Gradient Boosting (XGBoost) with the hyperparameters optimized through grid search were built. For estimation of model performance, repeated stratified k-fold cross-validation was used. The accuracy values of the generated models are in the range of 0.72 to 0.80. Analysis of feature importance indicates that mutations in distinct regions of SLC6 transporters are associated with an increased risk for pathogenicity. When applying the model on an independent validation set, the performance in accuracy dropped to averagely 0.6 with high precision but low sensitivity scores.

## Abstract 2

Predict SLC6 mutation clinical pathogenicity by calculating the amino acid descriptors in different ranges with rationalization analysis of the prediction.

## Graphical abstract

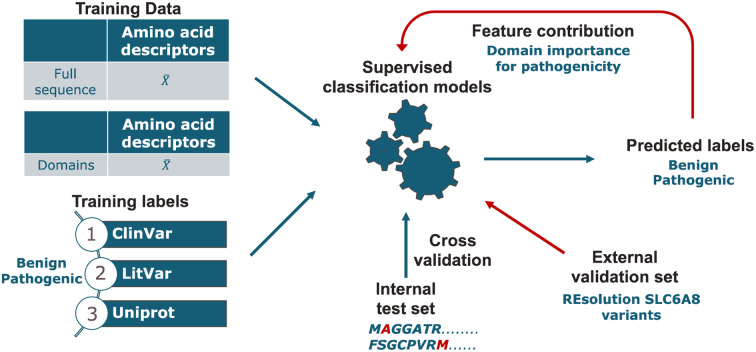

## 1 Introduction

The solute carrier (SLC) superfamily of human membrane transporters ranks among the largest membrane protein families in the human genome.^1^ It encompasses more than 400 proteins categorized into 66 families.^2,3^ This superfamily contains all membrane-spanning transport proteins that are not channels, ATP-driven pumps, aquaporins, porins of the outer mitochondrial membrane, or ATP-binding cassette (ABC) transporters.^4^

In this work, we focus on the SLC6 family, which is one of the most intensively studied ones.^5,6^ It is composed of 19 members and one additional pseudogene *SLC6A10*. Most members of this family play crucial roles in the trafficking of small amino acids or amino acid-like molecules such as serotonin, dopamine, norepinephrine, GABA, and creatine across membranes. Moreover, the whole SLC6 transporter family is characterised by regions of highly conserved sequences and structural folding patterns, such as the core transport region, where the sequence similarity between members can reach more than 60%. So far, only a few members have an experimentally determined structure, which shows a twelve-transmembrane domain (TMD) topology. They all share a ten helices motif that was observed in 2005 from a high-resolution X-ray crystallographic structure of a prokaryotic homolog, the Na^+^-dependent leucine transporter (LeuT) from *Aquifex aeolicus*.^7,8^ Since then, this LeuT-fold has been used to structurally and functionally describe this family as well as many other SLC families. Nonetheless, the understanding of the relationship between sequence, structure, and function dynamics keeps evolving.^9–11^

Genetic variations may lead to differences in disease susceptibility and absorption, distribution, metabolism, extrusion, and toxicity (ADMET) properties of drugs. Hence, missense mutations, those that translate into amino acids different from the prevalent ones in a certain population, are of special medical interest. Only when the missense mutation triggers significant changes during the protein function, also known as non-conservative missense mutation, clinical pathogenicity can occur. How those genetic variants in patients affect the function of SLCs is one of the fundamental questions on which the REsolution IMI consortium (https://re-solute.eu/resolution) is working on.^12^ As a cooperative work with many consortium partners, we focused on the development of mutation effect predictors that can provide a binary classification (pathogenic or benign) for the pathogenicity of mutations at SLCs, more specifically for the SLC6 family.

Continuous attempts based on *in silico* approaches have been made to identify functionally relevant human mutations with diverse *in silico* methods. These comprise, for instance, principal component analysis (PCA) of selected structure and sequence features, conventional machine learning architectures, artificial neural networks (NNs), as well as state-of-the-art natural language embeddings.^13–15^ Publicly available variant effect predictors (VEPs) share the disadvantage that they have not been specifically trained and tuned for the SLC superfamily. Consequently, their performances might be less satisfactory when applied to a special SLC subfamily.

Here, we present ProteoMutaMetrics modelling, a machine learning based pipeline focusing on the SLC6 family. This method is inspired by proteochemometrics modelling,^16^ where the binding pocket residues of a protein are taken into consideration together with small molecules to establish a predictive model for the structure–activity relationship of small molecules.^16^ Instead of focusing on the molecular activity of small molecules, we are interested in how the functionality of the protein is influenced by single point missense mutations. In the present method, the amino acid properties of the mutant and wild-type proteins were calculated and averaged on the full sequence range and domain-wise parts. These vectors were further used as input for machine learning algorithms to predict pathogenicity.

## 2 Results and discussion

### 2.1 Retrieved mutation data

**Fig. 1:**
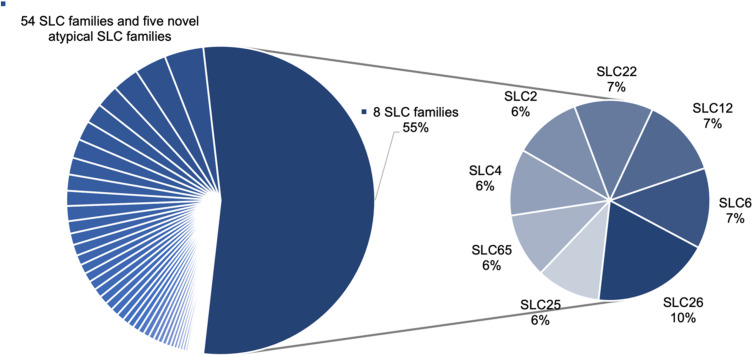
Mutation datapoint distribution on the SLC families. Eight families encompass 55% of the total number of mutations, while the remaining 45% are distributed across 54 SLC families and the five atypical SLC families.

We collected a total of 4383 mutation data points for the whole SLC superfamily. In total 67 SLC families have data including 62 SLC families named by the HUGO Gene Nomenclature Committee (HGNC) and five novel atypical SLC families.^17,18^ The mutation points are distributed very heterogeneously across the families, whereby eight families (Table 1) make up more than half (55%) of the data points (Fig. 1). No data was found for SLC families 66, 61, 50, and 48. Two of these (SLC61, SLC50) are currently all orphans according to the SLC family list from the RESOLUTE knowledgebase.^12,19^

**Table 1:** SLC families covering more than half of the mutation datapoints retrieved^a^

SLC family	Count of mutation datapoints	Count of SLC members with mutation data
SLC26	379	11
SLC6	259	17
SLC12	255	7
SLC22	254	18
SLC2	218	12
SLC4	214	8
SLC65	208	2
SLC25	206	38

a For each family, the total count of mutation data points and the count of SLC members in each family with data available from three databases after processing in KNIME are provided.

For the SLC6 family, a satisfactory coverage of the members (17 out of 19) and a fair amount of data points can be observed. In total, 259 mutation data points were retrieved, with 146 benign mutations and 113 pathogenic ones, including 21 different transcripts (Table S2†). Hence 259 mutated sequences were created. Together with the original 23 sequences, the final input dataset consists of 282 sequences.

### 2.2 Clusters from unsupervised approaches

**Fig. 2:**
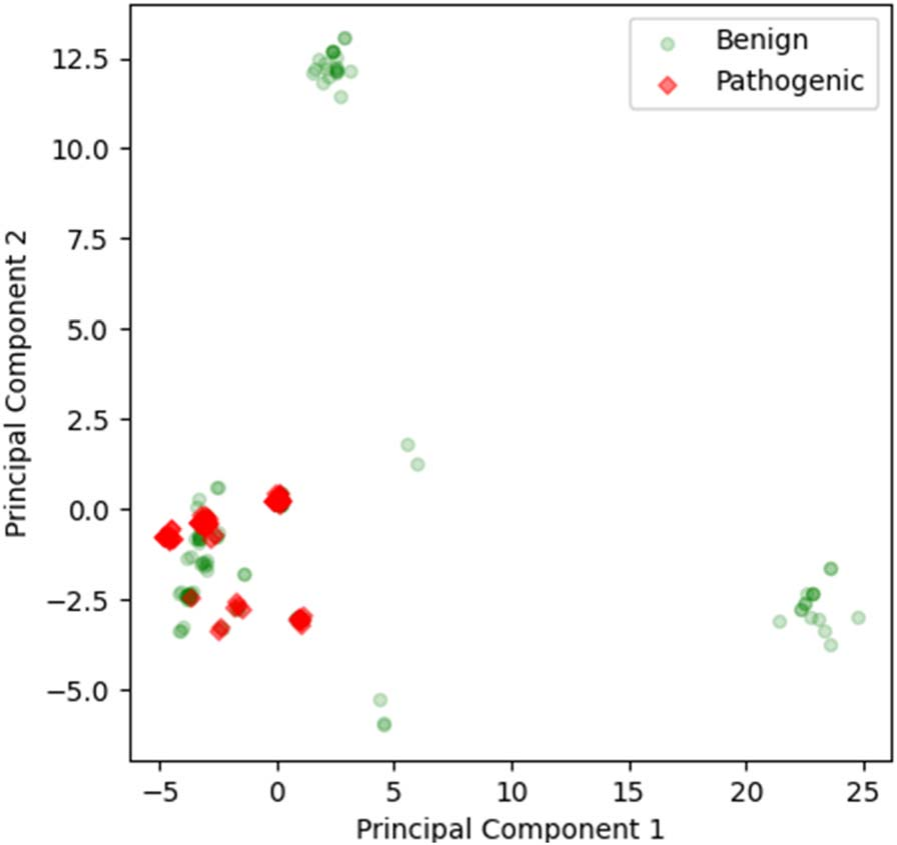
2D scatter plot of first two components from the full sequence based PCA analysis. The first two components cover 69% of the variance. The pathogenic data points are marked in red diamonds, while the benign ones are in green circles. The markers are transparent to avoid the overlay of the data and offer a better view of the enrichment of the clusters.

In the PCA analysis for the full sequence, four components are sufficient to represent more than 85% of the variance of the input, whereas in the domain-wise analysis, six components are needed to cover more than 85% of the variance (Fig. S1†). For the purpose of visualization, the first two components of full sequence based calculation were plotted in a 2D graph (Fig. 2) and the first three in a 3D graph (Fig. S2†). A few benign clusters can be spotted quite distant from the majority of the data points, which are located in the lower left corner and failed to be separated. This causes the shift of the 0 axes from the central position, which suggests that the data are not mean-centered.

**Fig. 3:**
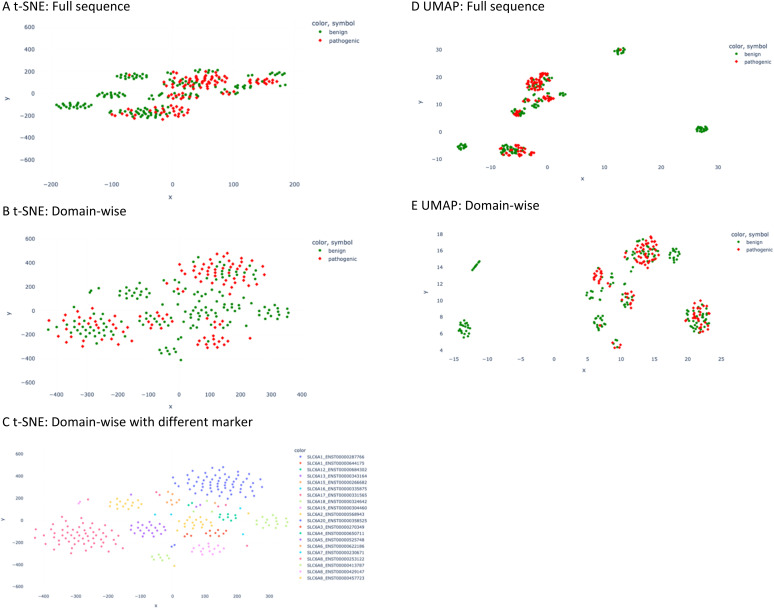
t-SNE and UMAP plot of the descriptor values. (A) t-SNE plot of full sequence calculation. The red diamond marker stands for pathogenic mutation and the green circle for benign one. (B) t-SNE plot of domain-wise calculation. (C) t-SNE plot of domain-wise plot is presented in a different marker scheme; namely different combinations of colors and symbols represent different SLC6 transcripts. (D) UMAP plot of the full sequence calculation. (E) UMAP plot of domain-wise calculation.

In such a data distribution scenario, linear dimension reduction techniques are not the preferred way for visualization of the data. Therefore, we applied two non-linear dimensionality reduction techniques – t-SNE and UMAP – for both the full sequence and the domain-wise representation. However, t-SNE and UMAP group pathogenic mutated sequences together with non-pathogenic ones (Fig. 3). In the majority of the clusters, pathogenic data points are not differentiable from benign ones. Nevertheless, a couple of clusters can be observed with benign labels only. Visually, the UMAP plot demonstrated a better separation between the clusters, which is in agreement with its better capability to preserve the global structure of the data when compared to t-SNE (Fig. 3D and E). However, when assigning the marker and color according to the SLC gene and transcript ID in the t-SNE domain-wise plot, a clear separation was achieved in most clusters (Fig. 3C).

### 2.3 Performance of supervised models

Subsequently, several supervised classification models trained on the pathogenicity label and the descriptor values were generated. They are based on the following machine learning architectures: Support Vector Machine (SVM), Logistic Regression (LR), Random Forest (RF), and Extreme Gradient Boosting (XGBoost). Optimized *via* grid search and then estimated through repeated stratified k-fold cross-validation (10 repeats, 10 folds), the performance of selected models is listed in Table 2. In the domain-wise calculation, the mean accuracy values of the generated models are in the range of 0.77 to 0.80, whereas in the full sequence representation, the values are between 0.72 and 0.80. Specifically, using averaged descriptor values on domains as input vectors enhances the performance of the SVM and LR models.

**Table 2:** Performance of the best models derived from grid search. (A) Calculating the average of amino acid descriptors over the full sequence. (B) Averaging amino acid descriptors over 12 domains. The performance is shown in five different statistical metrics with the respective standard deviation

	Accuracy↑	F1 score↑	Precision↑	Recall↑	ROC AUC↑
**A**					
SVM (RBF)	0.77(±0.07)	0.71(±0.09)	0.72(±0.11)	0.73(±0.13)	0.82(±0.09)
LR	0.72(±0.08)	0.59(±0.14)	0.70(±0.14)	0.54(±0.17)	0.76(±0.09)
RF	**0.80(±0.07)**	0.73(±0.07)	**0.77(±0.11)**	0.72(±0.14)	**0.88(±0.06)**
XGBoost	0.80(±0.06)	**0.74(±0.09)**	0.76**(**±0.11)	**0.73(±0.14)**	**0.88(±0.06)**

**B**					
SVM (poly)	**0.80**(**±0.08)**	**0.72**(**±0.11)**	**0.76**(**±0.13)**	**0.70**(**±0.14)**	**0.87**(**±0.08)**
LR	0.77(±0.09)	0.69(±0.12)	0.73(±0.14)	0.68(±0.16)	0.86(±0.07)
RF	0.78(±0.07)	0.70(±0.10)	0.73(±0.13)	**0.70**(**±0.14)**	0.86(±0.07)
XGBoost	0.77(±0.08)	0.69(±0.11)	0.73(±0.13)	0.67(±0.15)	0.87(±0.06)

When comparing the performance of different models, non-linear methods were observed to outperform linear ones. The sign of non-linearity in our data can also be visualized from the unsupervised clustering analysis, where the t-SNE 2D plot (non-linear dimensionality reduction) shows better separation than the PCA 2D plot. Moreover, in both full sequence and domain-wise representation, logistic regression, known to be suitable for linearly separable datasets,^20^ achieved the lowest performance with respect to the mean prediction accuracy, as well as to other statistical metrics (Table 2). The findings from the aforementioned supervised and unsupervised methods advocate the relevance of the non-linearity between the input data matrix (descriptor values) and the output (pathogenicity label).

By means of two different feature importance analysis techniques, we ranked the input features with the name of the domain and descriptor according to their contributions to the model performance (Fig. S3†).

**Fig. 4:**
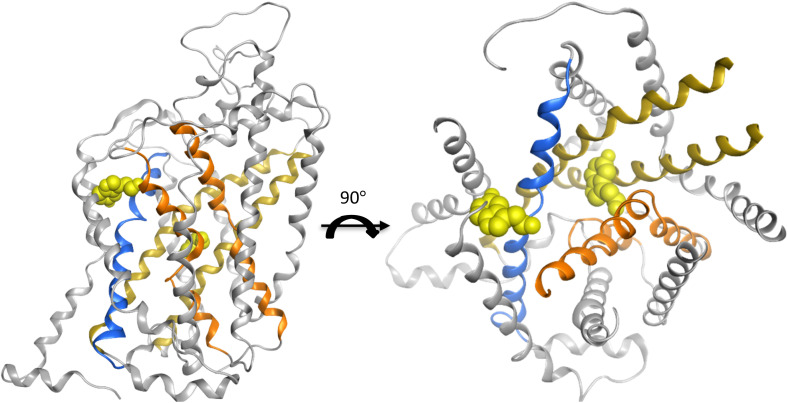
The crystal structure (PDB 7mgw) of SLC6A4 with the substrate serotonin co-crystalized in the central and the allosteric binding site. The helices three and eight are marked in dark yellow. The other two helices one and six, which are also related to substrate binding, are marked in orange. The helix ten engaged in the gating is in blue ribbon. The light-yellow sphere represents one molecule of leucine.

Out of all twelve domains, the top-ranked six domains/helices were taken from each plot of two feature importance analysis graphs for every model. Subsequently, the consensus features were selected as the final most contributing features for the performance of this model (Table 3). In this way, helix three and eight were captured to be the most favoured features regardless of the model architecture or the feature importance analysis technique. These two helices are well-characterized for their biological function in the translocation process of the substrate and hint towards disease-specific effects when mutated (Fig. 4).

**Table 3:** Feature importance analysis of each model with built-in attribute and permutation technique^a^

	Built-in feature importance	Permutation feature importance	Consensus features
SVM	Impossible for poly kernel	11, 8, 4, 9, 3, 6	
RF	12, 2, 8, 6, 3, 1	6, 12, 8, 1, 3, 5	12, 8, 6, 3, 1
LR	3, 2, 4, 6, 8, 10	8, 4, 2, 3, 6, 12	3, 2, 4, 6, 8
XGBoost	8, 10, 2, 12, 9, 3	3, 9, 6, 2, 8, 11	8, 2, 9, 3

a Consensus features were taken from both techniques for each model. As the poly kernel was used for hyperparameter tuning of the SVM, it is impossible to analyse the feature importance for this kernel with built-in attribute.

### 2.4 Domain importance interpretation for domain wise models

**Fig. 5:**
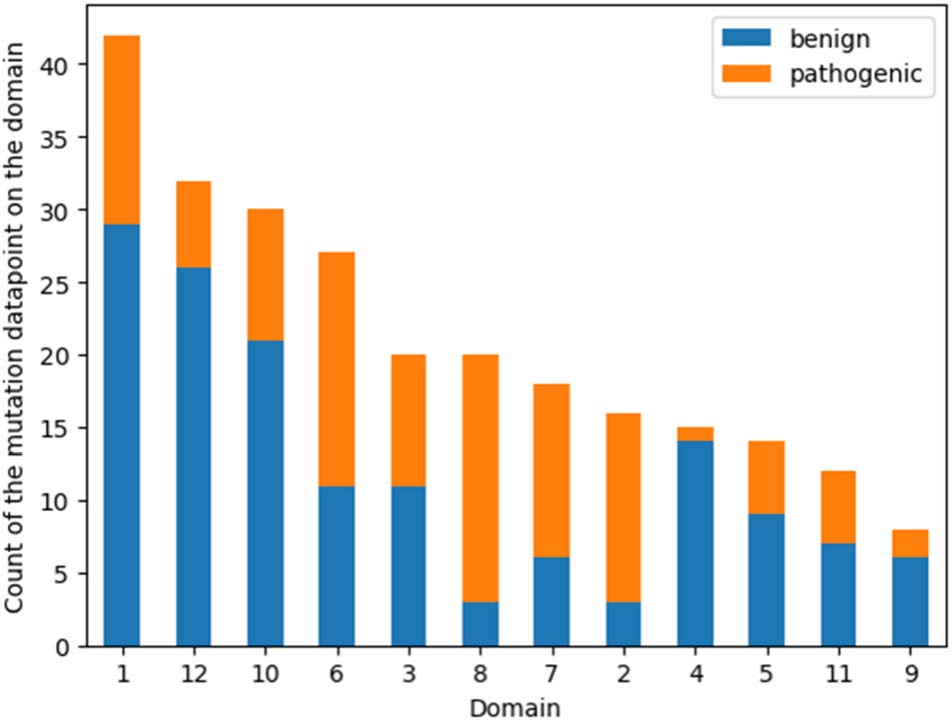
Bar plot of the number of mutations on each domain. The orange fragments stand for the pathogenic mutation data points and the blue for the benign ones.

To reveal a potential bias induced by the unbalanced distribution of point mutations in different domains, the count of benign and pathogenic mutations was plotted on the domain where their positions belong to. The helices ranking suggested by feature importance analysis was then compared with the ranking of the data amount (Fig. 5). Helices 1 and 12 already encompass 52% of all the mutation positions, yet these two domains were only picked by one model in the consensus feature importance analysis. This suggests that the amount of data does not significantly influence their ranking in the feature importance analysis. Moreover, the proportion of the benign and pathogenic mutation in each domain was visualized in Fig. 5. Helix 8 possesses a distinct higher fraction of pathogenic data points. This distribution was also observed in helix 2 and helix 7, while helices 3 and 6 show a more balanced fraction of pathogenic *vs.* benign mutations. This is somewhat contradictory to the result of the consensus feature importance analysis. While we do not have a clear explanation for this yet, its tempting to speculate that the descriptors used for building the models (z3 scales) are better able to capture the impact of mutations in areas involved in the substrate translocation pathway rather than in regions where mutations most probably affect folding and translocation.

### 2.5 Evaluation with REsolution in-house variant assay results

The best models with respect to their cross-validation performance were selected for further evaluation. To measure the robustness of these predictive models, all 30 SLC6A8 variants characterized with in-house assay results were taken as external validation set.

Four different pathogenicity label systems were listed for these 30 variants, namely the label from the database, the interpretation from the REsolution in-house assay result, the prediction from a combination of 15 VEPs^21^ and a consensus prediction of our models (Table 4).

**Table 4:** 30 variants of SLC6A8 collected and tested by REsolution consortium partners^a^

ID	Variant	Reported pathogenicity	Interpreted pathogenicity	Combined VEP prediction	Consensus model prediction
V1	K4R	Benign	Benign	**Benign**	**Benign**
V2	G26R	Unknown	Pathogenic	Benign	Benign
V3	Q114H	Unknown	Pathogenic	**Pathogenic**	Benign
V4	R207W	Pathogenic	Pathogenic	Benign	Benign
V5	F315I	Pathogenic	Pathogenic	**Pathogenic**	Benign
V6	G322W	Unknown	Pathogenic	**Pathogenic**	Benign
V7	N331K	Pathogenic	Pathogenic	**Pathogenic**	Benign
V8	T394K	Pathogenic	Pathogenic	Benign	**Pathogenic**
V9	P397L	Pathogenic	Pathogenic	**Pathogenic**	**Pathogenic**
V10	A404P	Pathogenic	Pathogenic	**Pathogenic**	**Pathogenic**
V11	L411S	Unknown	Pathogenic	**Pathogenic**	**Pathogenic**
V12	L412M	Unknown	Pathogenic	Benign	**Pathogenic**
V13	S417R	Unknown	Pathogenic	**Pathogenic**	**Pathogenic**
V14	G424D	Pathogenic	Pathogenic	**Pathogenic**	**Pathogenic**
V15	I457N	Unknown	Pathogenic	**Pathogenic**	**Pathogenic**
V16	G466R	Pathogenic	Pathogenic	**Pathogenic**	**Pathogenic**
V17	D474G	Pathogenic	Pathogenic	**Pathogenic**	**Pathogenic**
V18	S477L	Pathogenic	Pathogenic	**Pathogenic**	**Pathogenic**
V19	L484F	Unknown	Pathogenic	**Pathogenic**	Benign
V20	A487S	Unknown	Benign	**Benign**	**Benign**
V21	C491Y	Pathogenic	Pathogenic	**Pathogenic**	**Pathogenic**
V22	R502C	Unknown	Benign	Pathogenic	**Benign**
V23	D506N	Benign	Benign	**Benign**	**Benign**
V24	M510K	Pathogenic	Pathogenic	**Pathogenic**	**Pathogenic**
V25	V539I	Pathogenic	Benign	**Benign**	**Benign**
V26	T550S	Unknown	Benign	**Benign**	**Benign**
V27	V552L	Unknown	Benign	**Benign**	**Benign**
V28	W556S	Unknown	Pathogenic	**Pathogenic**	**Pathogenic**
V29	M560V	Unknown	Benign	**Benign**	**Benign**
V30	G561R	Pathogenic	Pathogenic	**Pathogenic**	Benign

a Correct predictions from combined VEPs and from the consensus model compared to the interpreted pathogenicity from assay results are marked in bold.

As shown in Table 4, the interpreted pathogenicity labels align well with the reported ones while complementing the “unknown” category with defined endpoints. When taking the interpretation as the ground truth for the mutation pathogenicity, the label balance is 73% to 27% for pathogenic and benign. The consensus of our models predicts 40% of these variants as pathogenic, which is close to the proportion of the pathogenic data points (44%) in the training set. In comparison, the prediction of a meta predictor composed of 15 different VEP approaches^21^ contains 63% of pathogenic labels. As the label of this external validation set is relatively imbalanced, the metrics F1 score was calculated for both predictions. For the consensus prediction from our model, the value is 0.73, for the combined VEPs it is 0.88. Interestingly, all the pathogenic predictions are correct from our model, while all the benign predictions are true for the combined VEPs.

**Fig. 6:**
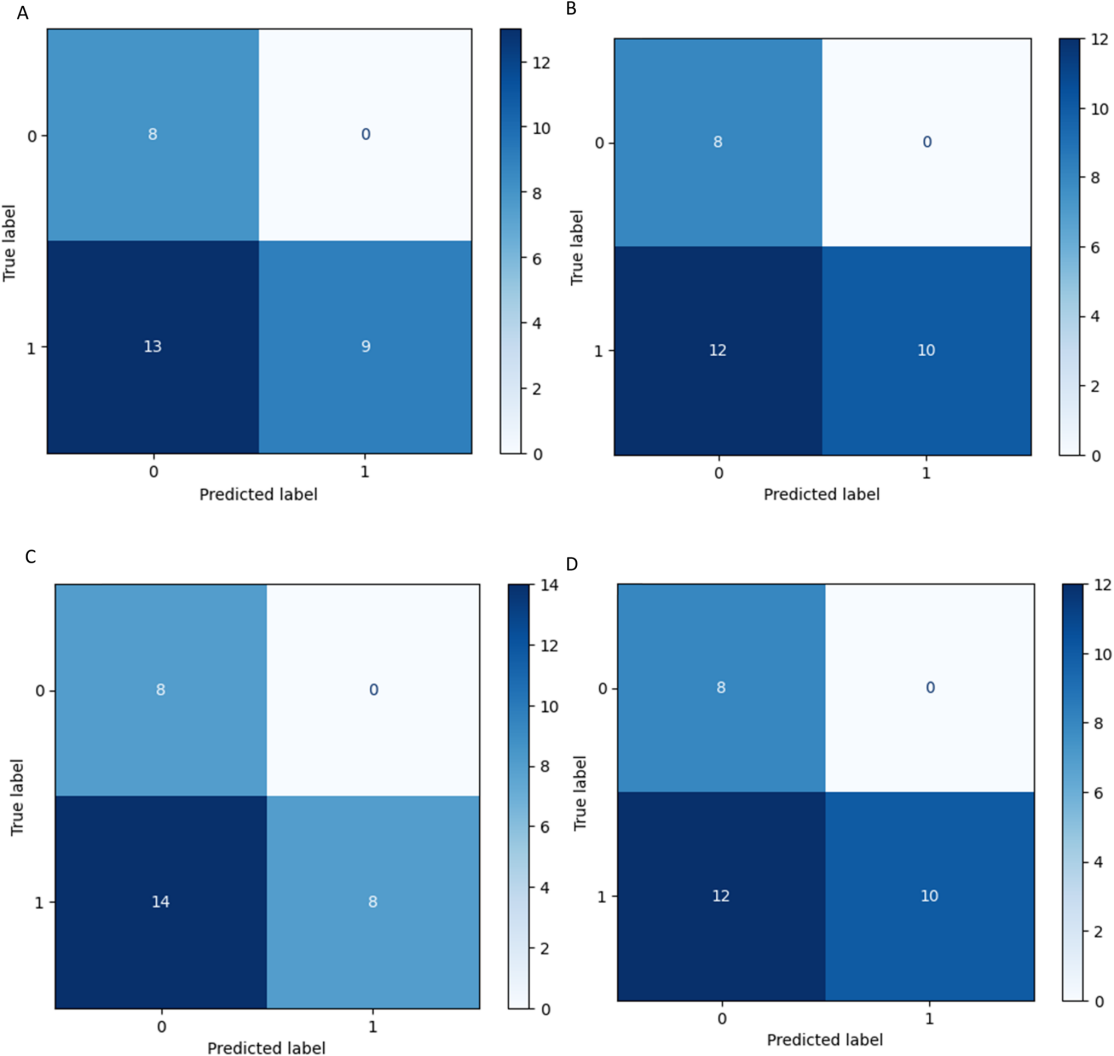
Confusion matrices of model performance. These models were selected from cross validation and then fit on the external validation set. (A) SVM (B) RF (C) LR (D) XGBoost.

Four statistical metrics are listed in Table 5 for the interpretation of each model's performance on the 30 variants. All four models reached accuracy values above 0.5, with the two tree-based algorithms RF and XGBoost reaching 0.6. The accuracy dropped from the range of 0.8 to 0.6 when applied to the external validation set. It is worth mentioning, that on all four confusion matrices (Fig. 6) there is no false positive prediction (*i.e.* a benign mutation predicted as pathogenic).

**Table 5:** The performance of four models selected from cross validation on the external validation set

	Accuracy↑	F1 score↑	Precision↑	Recall↑	ROC AUC↑
SVM	0.57	0.58	**1.00**	0.41	0.48
RF	0.60	0.62	**1.00**	0.45	0.53
LR	0.53	0.53	**1.00**	0.36	**0.82**
XGBoost	0.60	0.62	**1.00**	0.45	0.77
Consensus	**0.73**	**0.73**	**1.00**	**0.58**	0.72

In other words, all true benign variants are predicted by every four models as benign, and if the model predicts a mutation as pathogenic it is indeed pathogenic. However, a considerable number of pathogenic mutations are wrongly predicted as benign. This can also be captured by the high precision and low sensitivity scores of the models in Table 5.

When accessing the pathogenicity prediction from the very recently introduced approach AlphaMissense^22^ on this set, we found that they provide three classes for their prediction, namely pathogenic, benign, and ambiguous. In our study, considering the limited size of the external validation set, we included the “ambiguous” label utilizing the pathogenicity probability provided in the AlphaMissense_hg38 data set. In this way, the “ambiguous” label was converted into the binary pathogenic label system (pathogenic for probability > 0.5 and benign for probability < 0.5), so that the result is comparable with the result of our approach. For our external validation set, AlphaMissense reached an accuracy of 0.90, precision of 1, and sensitivity of 0.86.

## 3 Conclusion

The aim of the study was to develop an amino acid descriptor-based variant effect predictor inspired mutation effect predictor that is able to provide a binary classification for the pathogenicity of mutations in SLC transporters, more specifically for the SLC6 family. The cross-validation results support the hypothesis that it is possible to perform the desired prediction *via* machine learning with amino acid property descriptors.

For external validation, the models were used to perform predictions on the pathogenicity of a set of *in vitro* tested SLC6A8 single point missense mutations. While the performance was less satisfactory with respect to the overall accuracy value, models showed precision values of 1. This demonstrates that the models are capable of capturing signals for pathogenic mutations.

However, one factor that might influence the model performance on the external validation set is the label assignment. While its pathogenicity labels were assigned based on cellular location and functional assay results, the pathogenicity labels of our training data were extracted from a diverse set of public data sources.

While preparing the manuscript for submission, AlphaMissense, a mutation prediction from DeepMind was published. Thus, we tested its performance on our external validation set. Despite the fact, that their sophisticated architecture delivered considerable better statistical results, the training weight of the model is not provided and the model requires large computational resources to train or retrain. Nevertheless, the results for the external test set demonstrate that a global model trained on a very large data set outperforms a model for a transporter subfamily. However, there are still some aspects, where our approach differs from AlphaMissense. One aspect is that we have only two classes of labels, while AlphaMissense also provides “ambiguous” as one of the predicted classes. For the prediction on the 30 external validation data points, three “ambiguous” predictions were assigned as benign based on the pathogenicity probability for a proper comparison with our approach. Furthermore, in contrast to AlphaMissense, our approach allows assessment of the contribution of individual domains to the model.

In this study, the SLC6 family was used for demonstrating the possibility of predicting mutation pathogenicity with machine learning using averaged amino acid descriptor values. Due to data limitation, the external validation set is sorely from SLC6A8 which is well represented in the training data with respect to the data amount and the class distribution. Hence, in the scenario of some SLC6 members where less or no data was retrieved or the label is highly imbalanced, the performance of this approach will require further investigation.

## 4 Materials and methods

### 4.1 Collection of mutation data

To collect mutation data that can be used for further analysis and model building, we had the following prerequisites on the data sources. First, the sequence identifier is important to retrieve the exact sequence before the mutation took place (either the Ensembl canonical transcript or an alternative transcript). Second, clear amino acid position information is essential for locating the mutation. Last, curated clinical pathogenicity labels are vital for model training and validation. Following these prerequisites, three databases were selected to conduct this work, namely UniProt^23^ (https://ftp.uniprot.org/pub/databases/uniprot/current_release/knowledgebase/complete/docs/humsavar.txt, data retrieved on 25.02.2022), ClinVar^24^ (https://www.ncbi.nlm.nih.gov/clinvar/, data retrieved on 01.02.2022), and LitVar^25^ (https://www.ncbi.nlm.nih.gov/CBBresearch/Lu/Demo/LitVar/, data retrieved on 01.02.2022). As LitVar data are extracted from plain texts in biomedical literature, a threshold was set to the occurrence of a LitVar variant datapoint – namely count greater than 15 – to avoid unreliable records.

UniProt provides the pathogenicity label as “likely pathogenic or pathogenic”, “likely benign or benign”, and “uncertain significance” using the American College of Medical Genetics and Genomics/Association for Molecular Pathology (ACMP/AMP) terminology.^26^ This aligns well with the inbuilt curated pathogenicity label of ClinVar. In contrast, the labels in LitVar are quite heterogeneous, varying from “pathogenic”, and “benign”, to “risk factor” or “drug response”. The retrieved data were processed in KNIME^27^ to sort out the labels from different databases. First, any data point without pathogenicity label was excluded. Second, only labels comprising certain strings – “pathogenic or “benign” – are included. Third, in case of conflicts in the pathogenic label from different sources, the data point was deleted. Finally, redundant records from different sources were checked and merged into one.

We retrieved the sequence from Ensembl^28^ (Ensembl release 109) according to the given Ensembl transcript ID. In the absence of an identifier, the Ensembl canonical transcript of the corresponding gene ID was taken. For each case, we checked if the original amino acid of the mutation matched the one from the retrieved sequence on the annotated position. Only if the amino acids matched, it was replaced with the mutation.

### 4.2 Reference 3D structure and domain definition

Among all SLC6 transporters, 4 sub-members have at least 1 experimentally solved structure (Table S1†). After manually checking sequence coverage in the full wwPDB validation report for the coverage of residues with solved coordinates, one cryogenic electron microscopy (cryo-EM) structure of SLC6A19 with the PDB ID 6M17 was taken as the reference. It has a satisfactory resolution of 2.9 Å and a 93% modelled sequence coverage for in total of 654 amino acids. Another structure (PDB ID 6M18) released simultaneously within one publication^29^ showed comparable resolution and covered the same range of the sequence. However it contains a higher fraction (82%) of residues that have a poor fit to the EM map compared to the structure with PDB ID 6M17 (49%) according to their validation reports.

**Fig. 7:**
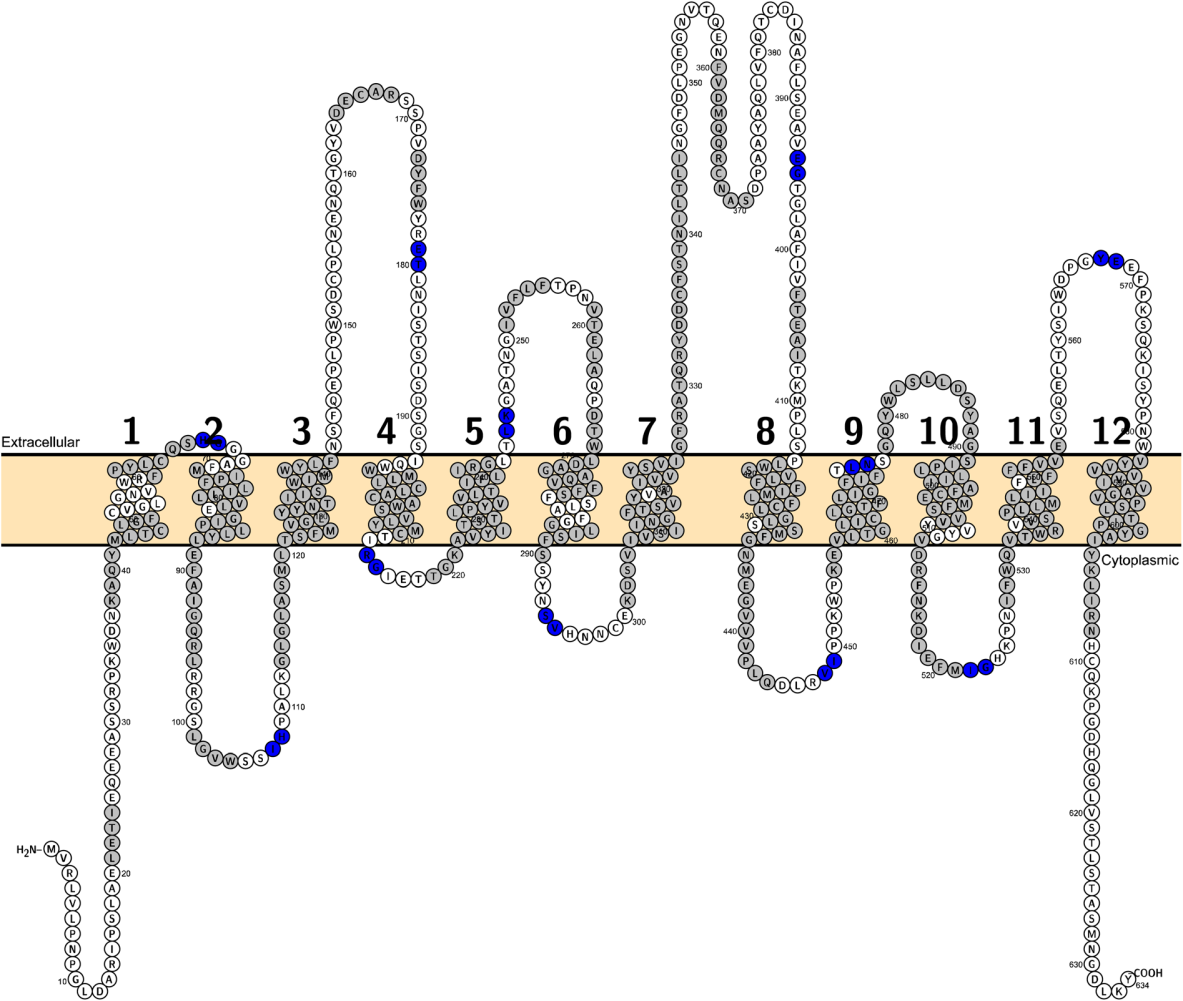
A topological visualisation of the hSLC6A19 sequence generated with Protter. The membrane is coloured in light orange. Residues in grey are the moieties with defined secondary structures. The cutting points lay in between each two residues in blue.

For the purpose of analyzing the domains, the whole sequence of this crystal structure was split into 12 domains^30^ (Fig. 7). The cutting points were determined manually through visual inspection using Molecular Operating Environment (MOE) software version 2022.02 (v2022.02; https://www.chemcomp.com). The cutting points for other SLC6 members were retrieved from the corresponding positions in a multiple sequence alignment (MSA). The MSA was created using the command line wrapper for MUSLE.^31^

### 4.3 Feature extraction

For every mutation in our dataset, the amino acid was alternated from the one in the wild type to the variant on the respective position. Subsequently, the averaged values of a selected set of amino acid descriptors were generated either on the full sequence or for each domain separately.

For full sequence calculation, we used a set of 75 amino acid descriptors derived from three types of properties of the amino acids: physicochemical, topological, and electrostatic properties.^32^ These descriptors can be grouped into 13 sets, namely Z-scales (Z3, Z5, and Z-Binned),^33^ ProtFPs (ProtFP-PCA3, ProtFP-PCA5, ProtFP-PCA8, ProtFP-Feature), T-scales,^34^ ST-scales,^35^ VHSE,^36^ MS-WHIM, FASGAI^37^ and BLOSUM.^38^ Their similarity and performance were elucidated and investigated in-depth by van Westen G. J. *et al.* in two benchmarking publications.^32,39^

However, when separating the sequence into 12 domains, calculating all 75 descriptors for 12 domains would result in 900 descriptor values, while the input mutation dataset comprises less than 300 data points. Thus, Z-scale descriptors were utilized for the domain-wise calculation. They cover the three aforementioned amino acid properties. Tentatively, Z1 can be linked to lipophilicity, Z2 to steric bulk, and Z3 to electronic properties.^33^ They are derived from a principal component analysis of a selected collection of experimentally derived physicochemical parameters.

Before proceeding with supervised as well as unsupervised approaches, the descriptor values were normalized using the StandardScaler function from scikit-learn.^40^

### 4.4 Unsupervised approaches

Principal component analysis (PCA),^41^ t-distributed stochastic neighbor embedding (t-SNE) plotting,^42^ and Uniform Manifold Approximation and Projection (UMAP)^43^ were conducted to gain a first view of potential clustering regarding the property changes due to mutations. In comparison to PCA, which preserves the global structure of the data on the maximum variated axes, t-SNE focuses on the neighborhood of the data location in the map. This allows the t-SNE to adapt to the underlying complex data by performing different transformations in different regions. Similar to t-SNE, UMAP is also a non-linear method for dimension reduction. However, the focus is UMAP is on the overall topology of the high-dimensional data with the aim to preserve both local and global structure in the data.^44^

### 4.5 Supervised approaches

Due to the small sample size, algorithms without exhaustive architectures were selected for the purpose of this study, namely Logistic Regression (LR), Support Vector Machine (SVM), Random Forest (RF), and Extreme Gradient Decent (XGBoost). For the first three methods, the scikit-learn implementation was utilized. In the case of XGBoost, a specialized Python package was taken from the Distributed (Deep) Machine Learning Community (DMLC) (https://xgboost.readthedocs.io/en/stable/python/, accessed 13 Oct).

Grid search was conducted to optimize the model hyperparameters. Repeated stratified k-fold cross validation (CV) with 10 repeats and 10 folds was computed for each hyperparameter set on each model.^45^ As our data distribution with respect to their pathogenicity labels was well balanced (pathogenic/benign = 40%/60%), data augmentation is not necessary in our case. Nevertheless, the stratified sampling in the CV process ensures that the label proportion is preserved in each training and test set.

The statistic metrics that were calculated to estimate the performance comprise the respective confusion matrix, accuracy, F1 score, precision, sensitivity, and the area under the receiver operating characteristic curve (ROC AUC) (Table 6). They were computed by defining the pathogenic mutation as the positive data point. Based on that, the predictions were classified as true positive (TP), false positive (FP), true negative (TN), or false negative (FN).

**Table 6:** The statistical metrics used for the assessment of model performance^a^

Metrics	Formula	Focus
Accuracy	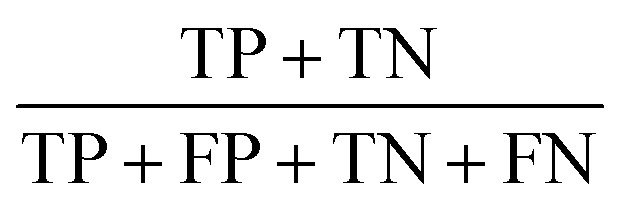	Overall effectiveness of the classifier
F1 score	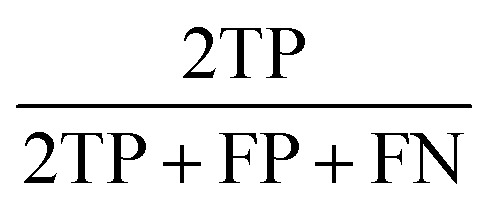	Combination of precision and sensitivity
Precision	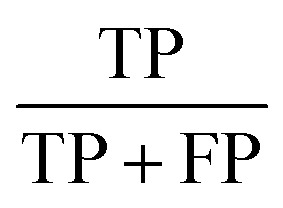	Class agreement of the data labels with the positive labels given by the classifier
Sensitivity	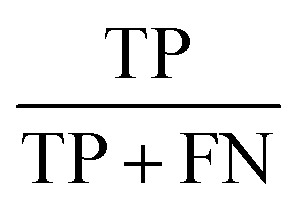	Effectiveness of a classifier to identify positive labels

a TP stands for true positive, TN for true negative, FP for false positive, and FN for false negative. As a positive datapoint, a pathogenic mutation was indicated.

### 4.6 Feature importance analysis

Feature importance analysis is a technique used to assess the contribution of each input descriptor to model performance. Considering the fact that tree-based models can be biased towards continuous data,^46^ we performed both built-in impurity-based and permutated feature importance analysis. Since we have a classification task, the scikit default Gini impurity was used.

For the permutation-based feature importance analysis, we shuffled each feature in and out 10 times from the prediction (*n*_repeats = 10) and calculated the change in accuracy. The best models out of hyperparameter tuning and cross validation were taken and fitted on the whole dataset.

### 4.7 External test set evaluation

As external test set, we used 30 SLC6A8 variants selected by REsolution consortium partners from various sources.^21^ As ten of them were already present in our data set, we moved them from the training to the test data set (variants: V1, V2, V3, V4, V16, V22, V23, V25, V26, V29; Table 4).

Importantly, all variants selected by the consortium were tested using an experimental activity assay. Both their cellular localisation and function were compared to the wild-type protein.^21^ The ground truth (interpreted pathogenicity) for the model training is summarized from in-house assay results (benign: as WT, slightly reduced; pathogenic: no response, reduced). These data points were applied as external validation set to assess each model's robustness.

A consensus prediction was generated by combining the best performing models from four different architectures. We used a minority rule to determine the consensus pathogenicity (*i.e.*, as long as one of the four models classifies a variant as pathogenic, the consensus prediction is determined as pathogenic). For the calculation of the statistic metrics of the consensus model, the accuracy, F1 score, precision, and sensitivity can be directly calculated from the binary classification. As for the ROC AUC score, the probability of the pathogenic class was taken from the greatest value from the four models.

## Conflicts of interest

There are no conflicts to declare.

## Supplementary Material

RA-014-D4RA00748D-s001
