# Supplementary material for: ProteoMutaMetrics: machine learning approaches for solute carrier family 6 mutation pathogenicity prediction
Source: RSC Adv. 2024 Apr 22;14(19):13083–94. doi: 10.1039/d4ra00748d (PMC11034476; doi:10.1039/d4ra00748d)
Supplement: RA-014-D4RA00748D-s001 [file RA-014-D4RA00748D-s001.pdf]

Electronic Supplementary Material (ESI) for RSC Advances.
This journal is © The Royal Society of Chemistry 2024

Supplementary material

Table S1 A summary of the SLC6 family with experimentally solved structures in PDB

SLC name	PDB ID	Method	Resolution	Sequence length	Sequence modelled	Percentage coverage
SLC6A1	7SK2	Cryo-EM	3.82 Å	578	532	92%
	6M17	Cryo-EM	2.9 Å	654	605	93%
	6M18	Cryo-EM	2.9 Å	654	605	93%
SLC6A19	6M1D	Cryo-EM	4.5 Å	654	605	93%
	7DWX	Cryo-EM	8.3 Å		-	
	7V61	Cryo-EM	3.2 Å	654	605	93%
	6VRH	Cryo-EM	3.3 Å	630	541	86%
	6VRK	Cryo-EM	4.1 Å	630	541	86%
	6VRL	Cryo-EM	3.8 Å	630	541	86%
	5I6X	X-ray	3.14 Å	549	544	99%
	5I6Z	X-ray	4.53 Å	549	544	99%
	5I71	X-ray	3.15 Å	549	544	99%
	5I73	X-ray	3.24 Å	549	540	98%
	5I74	X-ray	3.4 Å	549	542	99%
	5I75	X-ray	4.49 Å	549	540	98%
	6AWN	X-ray	3.62 Å	549	544	99%
	6AWO	X-ray	3.52 Å	549	544	99%
	6AWP	X-ray	3.8 Å	549	544	99%
SLC6A4	6AWQ	X-ray	4.05 Å	549	544	99%
	6W2B	X-ray	4.7 Å	549	544	99%
	6W2C	X-ray	6.3 Å	549	544	99%
	7LWD	Cryo-EM	3.65 Å	541	541	100%
	6DZZ	Cryo-EM	3.6 Å	540	540	100%
	7LI6	Cryo-EM	3.5 Å	539	539	100%
	7LI8	Cryo-EM	3.9 Å	539	539	100%
	7LI9	Cryo-EM	3.9 Å	539	539	100%
	7LIA	Cryo-EM	3.3 Å	539	539	100%
	7LI7	Cryo-EM	4.1 Å	537	537	100%
	7MGW	Cryo-EM	3.5 Å	537	537	100%
	6DZV	Cryo-EM	4.2 Å	537	537	100%

	6DZW	Cryo-EM	4.3 Å	537	537	100%
	6DZY	Cryo-EM	4.1 Å	537	537	100%
SLC6A9	6ZBV	X-ray	3.4 Å	577	539	93%
	6ZPL	X-ray	3.94 Å	576	518	90%

Note: The PDB ID was recorded together with the entity number of the respective SLC.

Table S2 The unique transcript ID and the SLC name of the collected mutation data

SLC name	Transcript ID	Canonical
SLC6A1	ENST00000287766	True
	ENST00000644175	
SLC6A2	ENST00000568943	True
SLC6A3	ENST00000270349	True
SLC6A4	ENST00000650711	True
SLC6A5	ENST00000525748	True
SLC6A6	ENST00000622186	True
SLC6A7	ENST00000230671	True
SLC6A8	ENST00000253122	True
	ENST00000413787	
	ENST00000429147	
	ENST00000457723	
SLC6A9	ENST00000372310	True
SLC6A12	ENST00000684302	True
SLC6A13	ENST00000343164	True
SLC6A15	ENST00000266682	True
SLC6A16	ENST00000335875	True
SLC6A17	ENST00000331565	True
SLC6A18	ENST00000324642	True
SLC6A19	ENST00000304460	True
SLC6A20	ENST00000358525	True

Note: Both canonical sequences and isoforms are included in this collection.

A

B

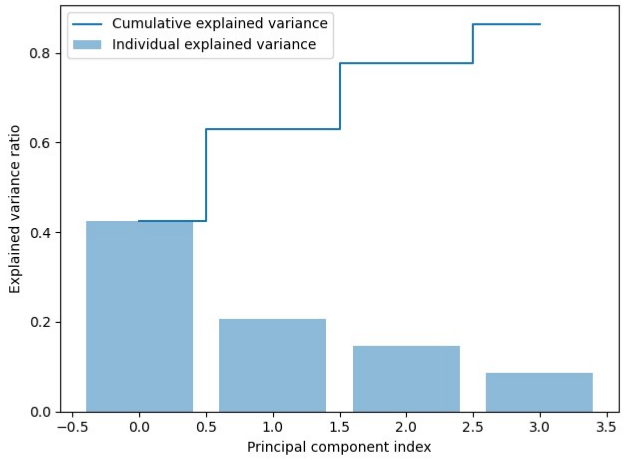

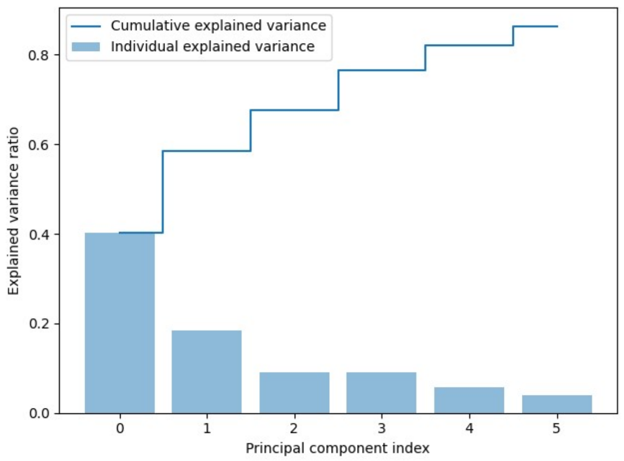

Figure S1 The principal component analysis. **A**. Full sequence calculation. **B**. Domain-wise calculation.

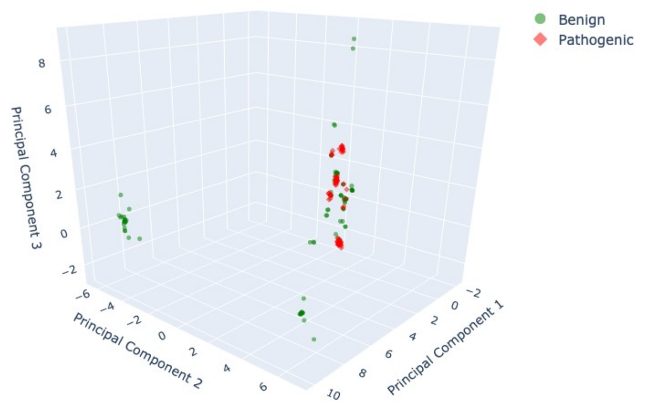

Figure S2 3D scatter plot of first three components from the full sequence PCA analysis

The pathogenic data point is marked in red diamond, while the benign one is in green circle.

A Random Forest

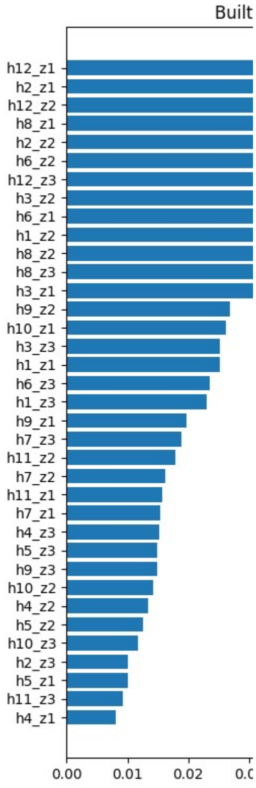

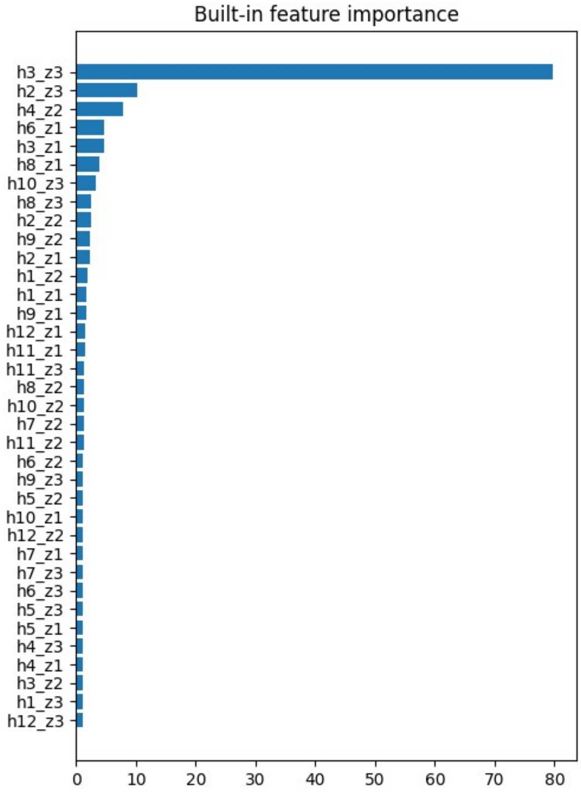

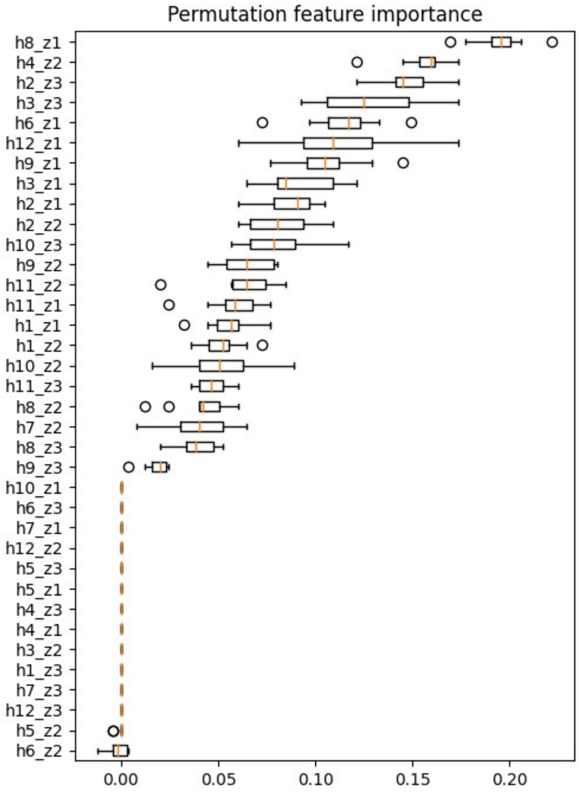

C Logistic Regression

B XGBoost

D Support Vector Machine

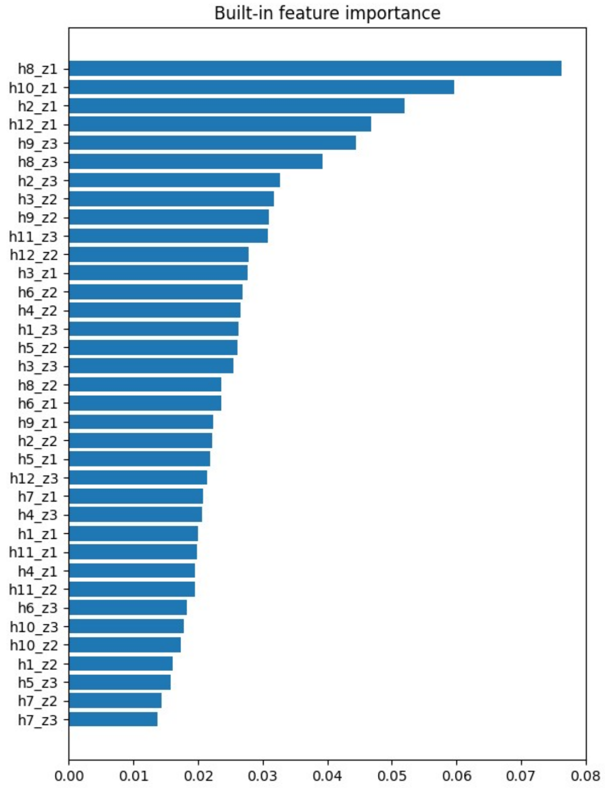

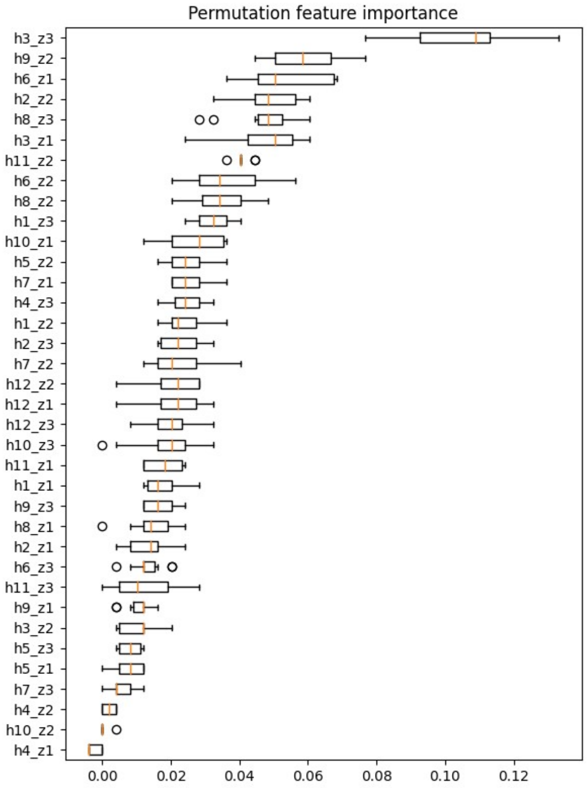

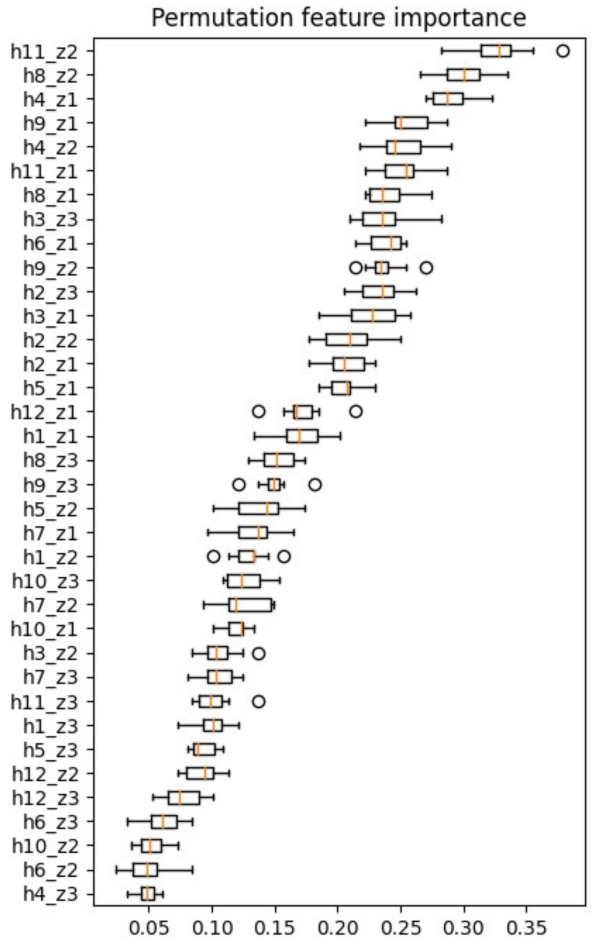

Figure S3 Built-in feature importance attributes horizontal bar plots and additional permutation feature importance box plots

A. Random Forest. **B**. XGBoost. **C**. Logistic Regression. **D**. Support Vector Machine. The features are named with the helix number and the descriptor name.
